# Is There a Role of Elevated CA 19-9 Levels in the Evaluation of Clinical Characteristics of Mature Cystic Ovarian Teratomas? A Systematic Review and Meta-analysis

**DOI:** 10.7759/cureus.6342

**Published:** 2019-12-10

**Authors:** Anastasia Prodromidou, Anastasios Pandraklakis, Dimitrios Loutradis, Dimitrios Haidopoulos

**Affiliations:** 1 Obstetrics and Gynecology, Alexandra Hospital, National and Kapodistrian University of Athens, Athens, GRC

**Keywords:** ca 19-9, biomarker, mature cystic teratoma, dermoid cyst, torsion, adnexal mass

## Abstract

The role of preoperative CA 19-9 levels in patients with ovarian mature cystic teratoma (MCT) and the association of elevated levels of the biomarker with patients’ and tumor characteristics were evaluated. Four electronic databases were searched for articles published up to September 2019. Trials that evaluated the significance of elevated CA 19-9 in patients with ovarian MCTs and publications with > 20 patients were considered eligible for inclusion. Seven studies that included 995 patients with an ovarian MCT who were evaluated with elevated (n = 364) or normal (n = 631) CA 19-9 levels were included. Mean tumor size was significantly increased in patients with elevated CA 19-9 levels (p = 0.038). The rate of ovarian torsion was significantly increased in the elevated CA 19-9 group (p = 0.04). The present study highlights the importance of CA 19-9 as a marker in the diagnosis of MCT, and a meta-analysis supports that it could raise a high degree of clinical suspicion of early recognition of torsion and early surgical management due to complications related to increased size. Nonetheless, the diagnostic value of CA 19-9 is still limited and CA 19-9 can still serve only as a supplementary diagnostic tool in patients with MCTs.

## Introduction and background

Adnexal masses remain a common problem in gynecology with a lifetime risk of surgery for a suspicious mass that ranges from 5% to 10% [1]. Among them, in pre-menopausal women, 90% of all ovarian masses are finally histologically proved of benign origin, whereas in post-menopausal women, malignancy is detected in 40% [2-3]. However, preoperative differentiation of ovarian tumors by imaging and blood tests are not specific and only surgical excision and histology can confirm the diagnosis. Mature cystic teratomas (MCTs), also known as dermoid cysts, are germ cell tumors and represent 60% of the most common benign ovarian tumors [4]. They are more frequently located in the gonads but they can also be detected in extragonadal sites, such as in the brain, omentum, Douglas pouch, and mediastinum [5-6]. In the majority of cases, MCTs are asymptomatic and the diagnosis is made incidentally on physical examination due to their size, in routine imaging of the lower abdomen, or during abdominal surgical procedures for other indications, such as cesarean delivery [7-8]. Symptoms can, on the other hand, be present in large MCTs due to pressure exerted on the surrounding structures or in cases of torsion or rupture in the abdominal cavity [9].

The tumor marker CA 19-9, also known as carbohydrate antigen 19-9 or cancer antigen 19-9, is a tetrasaccharide carbohydrate that has been proposed as a prognostic biomarker in patients with pancreatic cancer [10]. However, CA 19-9 has also been found either in other malignancies (hepatobiliary, colorectal, gastric, and lung cancer) or in benign diseases (pancreatitis, bile duct obstruction, liver cirrhosis, heart failure, Hashimoto’s thyroiditis, rheumatoid arthritis, and diverticulitis) [11-12]. Despite its common use as a tumor marker in the aforementioned non-gynecological diseases, there are also some cases in the literature of elevated CA 19-9 levels in ovarian cancer, as well as in benign ovarian masses [13]. Regarding benign gynecological diseases, single elevation in CA 19-9 levels has been detected in some cases of ovarian MCT. This has raised significant interest with regards to the role of CA 19-9 levels in the preoperative characterization of an ovarian mass as MCT and the respective avoidance of extensive unnecessary surgical procedures in women with benign masses. 

The objective of the present study was to accumulate the current knowledge on the role of preoperative CA 19-9 levels in patients with ovarian MCT and to evaluate the association of elevated levels of the biomarker with patients and tumor characteristics with special consideration in the potential contribution of the biomarker in the preoperative differential diagnosis among ovarian masses of benign and malignant origin.

## Review

Materials and methods

Search Strategy and Eligibility of Studies

The guidelines for the Preferred Reporting Items for Systematic Reviews and Meta-Analyses (PRISMA) were followed for the design of the present meta-analysis based on the authors’ predetermined eligibility criteria [14]. Three authors independently searched the literature (APr, APa, DH). No language restrictions were applied. All prospective and retrospective trials which evaluated the significance of elevated CA 19-9 in patients with ovarian MCTs and publications with > 20 patients were considered eligible for inclusion. Studies reporting outcomes of patients with elevated CA 19-9 and adnexal masses of other benign or malignant origin were excluded. Only comparative studies were recruited. Case reports, reviews, and animal studies were additionally excluded from the tabulation. Each author independently reviewed the literature; the discrepancies during the data collection were then resolved by consensus of all authors.

Literature Search and Data Collection

**Figure 1:**
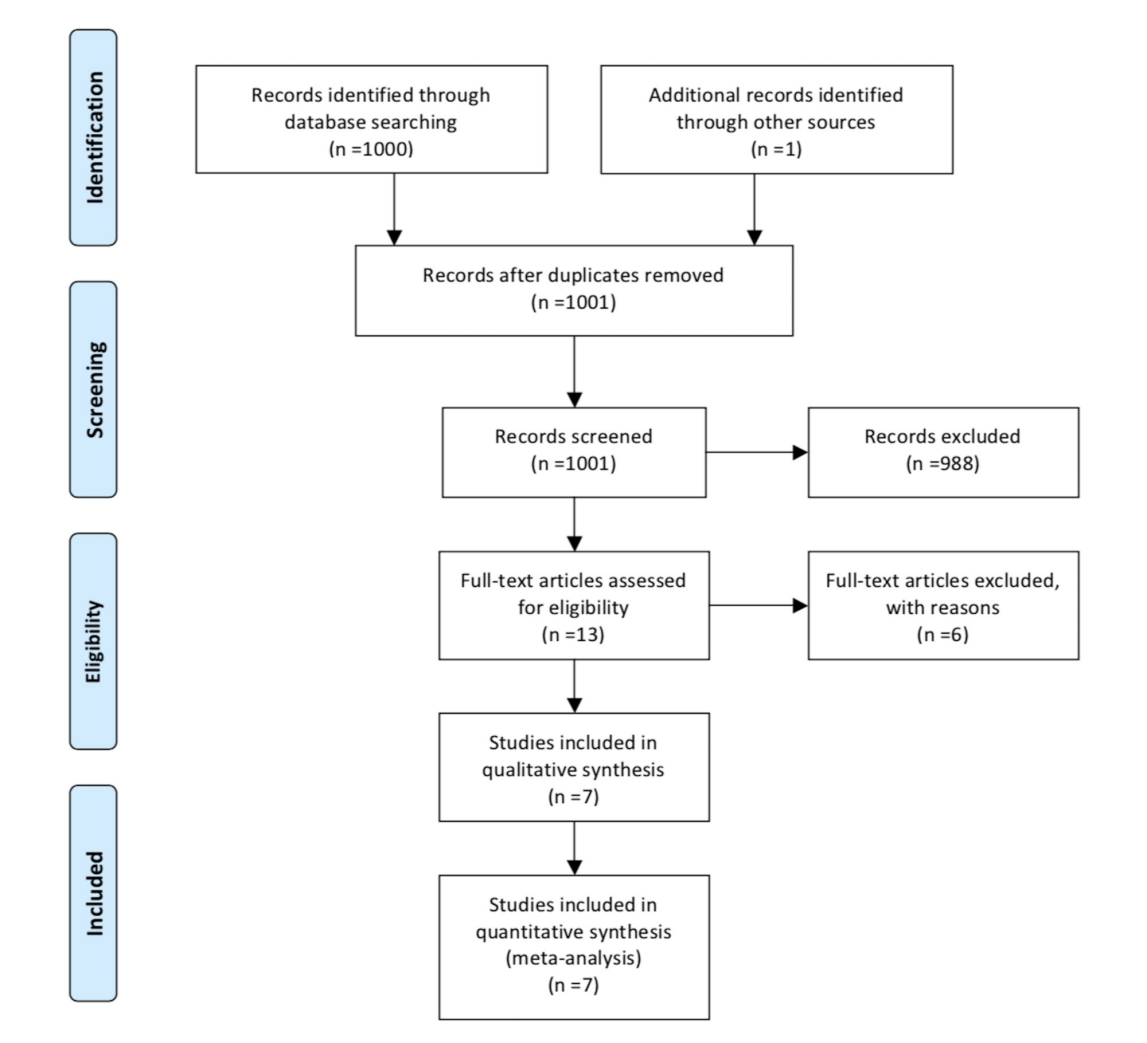
Search flow diagram

A systematic search of the Medline (1966 - 2019), Scopus (2004 - 2019), Google Scholar (2004 - 2019), and Clinicaltrials.gov databases for articles published up to September 2019 was performed. Articles included in the reference lists in the studies which were retrieved in full text were additionally systematically searched for relevant articles in the field. The performed search included the words “mature cystic teratoma,” “CA 19-9,” “tumor markers,” “ovarian teratoma,” “torsion,” “adnexal mass,” and “dermoid cyst” (Figure 1).

Data on patient characteristics included age, serum CA 19-9 level, tumor size, side of the lesion, presence of torsion, histological components of the mass and co-existence of elevated CA-125 according to each group of either normal or elevated CA 19-9 levels.

Quality Assessment

The quality of all the included studies was assessed using the Methodological Index for Non-Randomized Studies (MINORS) [15]. The MINORS scale was used because all of the studies included in our meta-analysis were non-randomized. Two authors independently performed the procedure.

Statistical Analysis

Statistical meta-analysis was performed using the RevMan 5.3 software (The Nordic Cochrane Centre, Copenhagen, Denmark). Confidence intervals (CI) were set at 95%. Mean difference (MD) and odds ratios (OR) were used in the analysis. The results were calculated using the DerSimonian-Laird random effect model (REM) revealing significant heterogeneity in the methodological characteristics of the included studies [16]. The cut-off for statistical significance was set at p < 0.05. Mean values and standard deviations were calculated according to the equations proposed by Hozo et al. when not provided by the studies [17]. Publication bias was not tested due to heterogeneity of the included studies, which is a confounder that may influence the methodological integrity of these tests.

Definitions

The cut-off value for CA 19-9 was 37 U/mL. Serum levels above this cut-off value were defined as elevated CA 19-9.

Results

Excluded Studies

Six studies were excluded from tabulation and analysis after reading the full text [13, 18-22]. Among them, three were excluded due to insufficient data [18-21]. Finally, Sagi-Dain et al. included patients with benign diseases and did not report separate outcomes for those with MCT whereas Wang et al. enrolled only patients with torsion in their study group and were both excluded [13-22].

Included Studies

A total of seven studies that included 995 patients with ovarian MCT who were evaluated with elevated or normal serum CA 19-9 levels were finally included in the present meta-analysis [23-29]. Among them, an increased serum CA 19-9 level was detected in 364 patients, whereas in the remaining 631 patients, normal CA 19-9 levels were measured. The analyzed indices were tabulated in two structured tables as follows: methodological characteristics of the included studies (Table 1) and characteristics of the included patients (Table 2).

**Table 1:** Characteristics of the Included Studies MCT: mature cystic teratoma; MINORS: Methodological Index for Non-Randomized Studies; N/A: not available; RS: retrospective

Author/Ref. #	Country	Type of study	MINORS	Inclusion criteria
Yesilyurt et al. [23]	Turkey	RS	11	Age ≤ 35 years; laparoscopic surgery; no pregnancy; no concomitant pelvic pathology, such as myoma or endometriosis; no malignant transformation of teratoma or another type of malignant lesion; no severe renal or hepatic disease
Frimer et al. [24]	USA	RS	15	Histological diagnosis of MCT; available data for tumour markers; no other incidental pathology
Cho et al. [25]	Korea	RS	14	Pathologically confirmed MCT; available data for tumour markers
Cengiz et al. [26]	Turkey	RS	15	No emergent cases; available data for tumour markers
Kyung et al. [27]	Korea	RS	15	No cases with ovarian or uterine diseases
Üstünyurt et al. [28]	Turkey	RS	15	No malignant transformation
Dede et al. [29]	Turkey	RS	16	N/A

**Table 2:** Patients Characteristics (Elevated vs Normal CA 19-9) ^a^Median (range); ^b^Mean ± SD (standard deviation) Bil: bilateral; L: left; N/A: not available; R: right

Author/Ref #	Patient No	Serum level	Elevated CA 19-9 N (%)	Age (years)	Tumor size (cm)	Side (Bil/L/R)	Torsion (N)	Histological components	Elevated CA-125
Yesilyurt et al. [23]	18 vs 59	N/A	18/77 (23.4%)	N/A	N/A	Bil 4/18 vs 11/59	N/A	N/A	N/A
Frimer et al. [24]	52 vs 87	131.3 (38.2-11 435)^a^ vs 11.2 (1-35.7)^a^	52/139 (37.4%)	42.6±15.5^b^ vs 41.2±13.7^b^	7.9±3.6^b^ vs 7.5±4.2^b^	Bil 6/52 vs 6/87	N/A	N/A	N/A
Cho et al. [25]	105 vs 134	N/A	105/239 (44%)	N/A	8.53±3.84^b^ vs 6.95±3.97^b^	Bil 19/105 vs 18/134	N/A	Fat: 55/105 vs 27/134; Calcification: 32/105 vs 31/134; Soft tissue: 30/105 vs 38/134; Solid portion: 7/105 vs 9/134; Septation: 10/105 vs 13/134	11/105 vs 9/134
Cengiz et al. [26]	32 vs 78	34.72±12.23^b^ vs 37.33±12.88^b^	32/110 (29%)	N/A	<4 cm 2 vs 13; 4-10 cm 27 vs 58; >10 cm 3 vs 7	Bil 0 vs 5 R 18 vs 44 L 14 vs 29	5 vs 9	Sebum: 25 vs 52; Hair: 21 vs 48; Keratin: 2 vs 20; Cartilage: 4 vs 14; Teeth: 3 vs 1	N/A
Kyung et al. [27]	52 vs 111	82.2 (37.2-575.0)^a^ vs 15.2 (4.0-36.8)^a^	52/163 (32%)	33.5±12.5^b^ vs 33.75±8.17^b^	9.5±3.5^b^ vs 9.25±3.17^b^	Bil 10 vs 23 R 28 vs 50 L 14 vs 38	11 vs 10	N/A	N/A
Üstünyurt et al. [28]	74 vs 113	193.6±247.7^b^ vs 11.5±8.9^b^	74/187 (39.6%)	36.9±13.4^b^ vs 37.5±12.6^b^	8.8±4.5^b^ vs 7.1±4.5^b^	Bil 9/74 vs 9/113	N/A	N/A	33/74 vs 13/113
Dede et al. [29]	31 vs 49	246.8±243.5^b^ vs 21.7±30.7^b^	31/80 (38.8%)	31.9±12.5^b^ vs 33.6 ±10.9^b^	10.1±6.7^b^ vs 5.9±2.4^b^	Bil 16/31 vs 6/49	N/A	N/A	N/A

Quality Assessment

The quality assessment using the MINORS scale showed that the included studies were methodologically adequate with low heterogeneity regarding their quality, providing a mean score of 14.4 (SD: 1.5) and a median score of 15 (range: 14 - 16) (Table 1).

Elevated CA 19-9 and Patient/Tumor Characteristics

**Figure 2:**
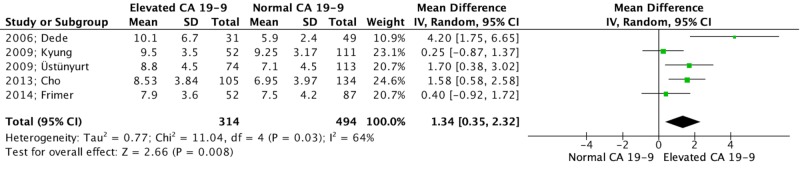
Forest plot depicting tumor size (cm) [29, 28, 27, 25, 24] CA: carbohydrate antigen; CI: confidence interval; df: degrees of freedom; IV: independent variables; I^2^: included^2^; SD: standard deviation; Z: Z-value (coefficient (B or D) divided by its standard error)

The total proportion of patients with elevated CA 19-9 was 36.6% (364 out of 995 patients) and ranged from 23.4% to 44% among the included studies. No difference was found in the mean age of patients among women with normal and elevated CA 19-9 levels (209 and 360 patients, respectively = 569 total patients, MD: -0.30 years, 95% CI: -2.54 to 1.93, p = 0.79). In patients with elevated CA 19-9 levels, tumors with a significant increase in mean size were detected compared to those with normal CA 19-9 values (314 vs 494 patients, respectively = 808 total patients, MD: 1.34 cm, 95% CI: 0.35 to 2.32, p = 0.038) (Figure 2).

**Figure 3:**
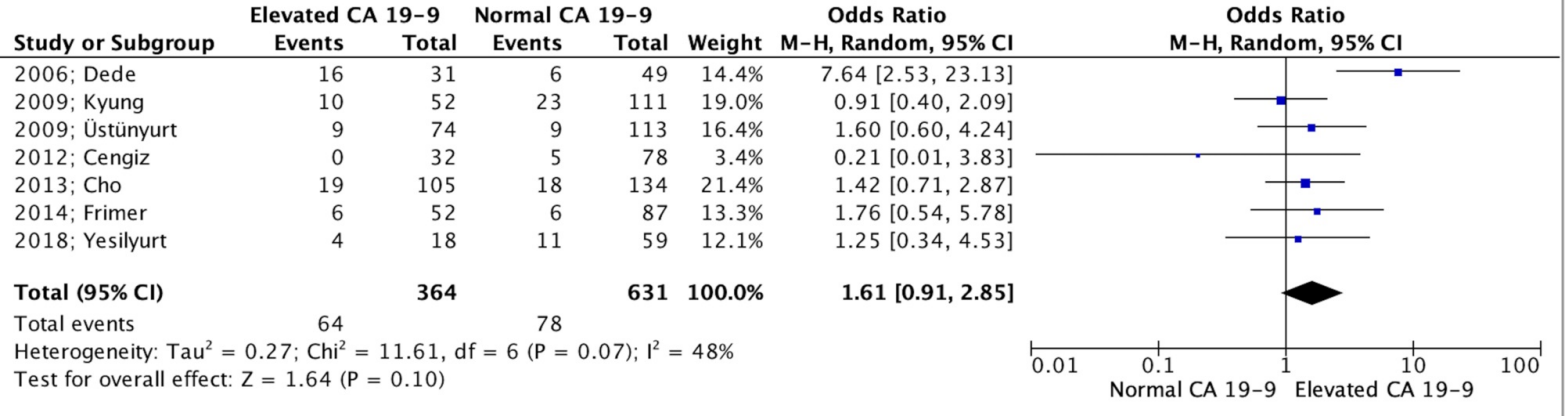
Forest plot depicting bilaterality rates [29, 28, 27, 26, 25, 24, 23] CA: carbohydrate antigen; CI: confidence interval; df: degrees of freedom; I^2^: included^2^; M-H: Mantel-Haenszel; Z: Z-value (coefficient (B or D) divided by its standard error)

This was not observed in the rates of bilaterality nor in the incidence of left and right-sided MCTs in which all three parameters did not differ among the two groups (995 patients, OR: 1.61, 95% CI: 0.91 to 2.85, p = 0.10; 273 patients, OR: 0.93, 95% CI: 0.51 to 1.70, p = 0.82; and 273 patients, OR: 1.24, 95% CI: 0.74 to 2.08, p = 0.42, respectively) (Figure 3). 

**Figure 4:**
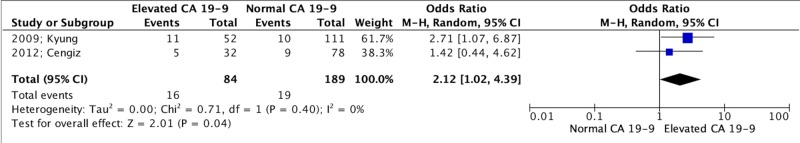
Forest plot depicting torsion rates [27, 26] CA: carbohydrate antigen; CI: confidence interval; df: degrees of freedom; I^2^: included^2^; M-H: Mantel-Haenszel; Z: Z-value (coefficient (B or D) divided by its standard error)

On the other hand, the rate of ovarian torsion was significantly increased in the elevated CA 19-9 group (273 patients: OR 2.12; 95% CI 1.02 to 4.39; p = 0.04) (Figure 4).

Rates of concomitant elevated CA-125 levels were not found to be different among the two groups (426 patients: OR 3.27; 95% CI 0.88 to 12.10; p = 0.08). Finally, the relation between elevated CA 19-9 levels and the type of histological components in the MCTs was also identified by two of the included studies. In that setting, Cho et al. detected that only fat components in MCT was positively correlated with elevation in CA 19-9 levels (p < 0.0001), while Cingez et al. found significantly less keratin and more teeth as part of MCT in patients with elevated CA 19-9 (p = 0.021 and p = 0.039, respectively) [25-26].

Elevated CA 19-9 and Differentiation From Malignancy

Outcomes regarding the comparison of elevated CA 19-9 in patients with MCT and ovarian cancer were available only by Cho et al. who showed that despite the fact that simultaneous elevation of CA 19-9 and CA 125 was more prevalent in patients with malignancy, single elevation of CA 19-9 was more frequently detected in MCTs compared to malignancies [25]. Additionally, Frimer et al. observed significantly elevated preoperative imaging with computed tomography (CT) in patients with an ovarian mass and elevated CA 19-9 which could indicate an increased suspicion of malignancy [24]. However, they suggested that for a patient with an ovarian mass with ultrasonographic characteristics of MCT and elevated CA 19-9 without other elevated markers, a further preoperative investigation would not have an additional diagnostic impact.

Discussion

The present study aimed to evaluate the association of the CA 19-9 antigen with the special characteristics of ovarian MCTs. The meta-analysis revealed a significantly increased MCT size in patients with elevated CA 19-9. Torsion was more prevalent in the elevated CA 19-9 group. On the other hand, no significant relation was found among patients’ age, site of the lesion, bilaterality, and simultaneous elevation of CA-125 in patients with elevated CA 19-9.

Despite significant progress noted in synchronous imaging techniques, preoperative differential diagnosis of adnexal masses remains challenging. Although the preoperative diagnosis of an ovarian mass is considered of high importance for the management of the disease, the histological examination of the mass remains the most reliable method to set the diagnosis. Mature cystic teratomas are the commonest surgically treated ovarian pathology in premenopausal women and are commonly incidentally diagnosed during ultrasound (US) examination for other indications [30]. In this age group, preoperative diagnosis was of critical importance so as to avoid over-treatment and to preserve the fertility of the patient.

CA 19-9 was firstly introduced in 1979 when Koprowski et al. described a monosialoganglioside antibody connected with an antigen found in the cultures of cells of colorectal cancer specimens [31-32]. This antigen was then found in epithelial cells of various malignancies whether of the gastrointestinal (GI) origin or not. Hence, CA 19-9 levels have been used as a tumor marker in various malignancies, including GI, pancreatic cancer, and cholangiocarcinoma. Furthermore, elevated CA 19-9 levels have been detected in benign conditions, especially those related to cholestasis and biliary pathology [33]. However, elevated serum CA 19-9 levels have also been reported in cases of gynecological diseases, but the exact role of the marker in differentiating malignant from benign conditions, as well as in the prognosis of the disease and the correlation with elevated CA-125 levels, remains elusive [34]. Elevated CA 19-9 levels have been detected in patients with mucinous histological types of ovarian masses and the marker was formerly considered of clinical importance in diagnosing ovarian malignancies, specifically when it was combined with elevated serum CA-125 [35-36]. However, according to recent studies in this field, the role of CA 19-9 in differentiating malignant from benign conditions, as well as in identifying the mucinous type of ovarian malignancy, remains controversial [13]. To that end, Sagi-Dain et al. noted significantly higher CA 19-9 levels in patients with an MCT than those with ovarian carcinoma [13]. This is in accordance with the findings of the present study, which revealed a higher prevalence of single elevated CA 19-9 in MCT cases compared to ovarian cancer [25]. Consequently, one could argue that in cases of single elevation of CA 19-9, suspicion of malignancy is lower and further preoperative investigation for malignancy other than US performance will not enhance the diagnostic procedure. Even though ultrasonography is the most common imaging technique for preoperative identification of MCT, ultrasonographic features are not always specific to preoperatively diagnose an ovarian mass as an MCT [37]. In cases of non-diagnostic US, computed tomography (CT) can accurately detect the fatty and calcified components (teeth) of the MCT with a sensitivity that ranges between 93% - 98% for the diagnosis of MCT [37]. Additionally, a CT scan can more specifically identify the correlation of the mass with the surrounding tissues. Similarly, magnetic resonance (MR) could also be considered as an alternative imaging tool when the US findings are inconclusive with respective high sensitivity in the detection of fat and calcifications [38]. 

The rates of elevated CA 19-9 in the present study were 37% which were comparable to those reported by other studies [19, 39]. More specifically, in the study by Kataoka et al., the proportion of patients with elevated CA 19-9 was 39%, whereas 23% and 6% of the included patients were found with CA 19-9 levels > 100 U/mL and > 500 U/mL, respectively [19]. The same authors reported the highest value of the biomarker in the literature which reached the level of 25,590 U/mL [19].

The exact pathophysiologic pathway of CA 19-9 elevation in patients with MCT remains elusive. Ito et al. described the detection of CA 19-9 in the immunochemistry of bronchial glands and bronchial mucosa of MCT specimens, as well as elevated CA 19-9 levels in the fluid contained in the MCT [40]. This suggests the potential mechanisms through which CA 19-9 was detected elevated in the blood, either through secretions of the antigen from bronchial glands direct to the bloodstream or in case of leakage through the absorption from the surrounding tissues. Accordingly, in patients with a ruptured endometrioma, it has been proposed that fluid leakage from the endometriotic cyst and the subsequent exchange among peritoneal fluid and serum could result in elevated serum CA 19-9 levels [41]. This could also explain the significantly elevated CA 19-9 in cases of torsion; one could hypothesize that necrosis, as a consequence of torsion, could cause dissemination of the cystic component of the MCT and can raise the serum CA 19-9 levels. This is in accordance with the findings of the present study which indicated a significant increment in torsion rates in patients with high CA 19-9 levels. Furthermore, according to a retrospective study by Wang et al., CA 19-9 levels were significantly higher in patients with torsion when compared to those without torsion of MCT (p < 0.001) [22]. According to the findings of the present study, patients with larger tumors were more likely to present with elevated CA 19-9 levels. Tumor size plays an important role in MCTs since larger tumors are frequently related to complications, including the risk of rupture and torsion, as well as the potential of malignant transformation [42]. Thus, an elevation in serum levels of CA 19-9 would supplement the imaging findings in early detection and prediction of the aforementioned complications. Additionally, elevated CA 19-9 levels could be considered as a potential indicator for early surgical intervention due to the higher risk of torsion, as well as the incidence of larger size. The association of elevated CA 19-9 levels with the components of the MCT was also evaluated in the present study, indicating that the fat and teeth components were significantly elevated in patients with high CA 19-9 when compared to those with normal levels of the biomarker. Interestingly, even though an endodermal component would be expected to be identified in cases of elevated CA 19-9 based on the suggested mechanism of secretion from bronchial mucosa and glands, the included studies indicated mesodermal and ectodermal components as the most frequent in high CA 19-9 levels [25-26]. However, the exact pathophysiologic pathways could not be clearly explained based on the existing literature and further larger studies are needed to identify the exact prevalence of each component in MCTs and the potential pathways of CA 19-9 elevation based on each of the three components. 

In the present study, we sought to perform a meticulous review of the current literature. To the best of our knowledge, this is the first meta-analysis in the literature, which presents a cumulative report and evaluation on the impact of CA 19-9 biomarkers in predicting the clinical characteristics of MCTs in females. Data restrictions were avoided with the intent to eliminate data losses which consists of a significant strength of the present study. Nonetheless, in order to evaluate our outcomes, the factors which potentially eliminate our findings, should be addressed. The true prevalence of patients with MCT and concomitant elevated CA 19-9 could not be precisely reached and data concerning its pathophysiology and clinical characteristics is limited. Additionally, the normalization of elevated CA 19-9 levels postoperatively was under-reported by the included studies. Furthermore, screening for the presence of concomitant GI pathology, which would be a confounder in the estimation of elevated CA 19-9 levels, was only stated by one of the included studies. Particularly, Ustunyurt et al. presented no simultaneous GI pathology in any of the recruited patients and suggested no benefit of further investigation of the GI tract in patients with elevated CA 19-9 and US characteristics suggestive of an MCT [28]. The limited number of studies that are available in the literature, along with the small number of the included patients, cannot allow us to draw any safe conclusion on the specific role of CA 19-9 in MCTs. Finally, the significant heterogeneity of them, along with the fact that some parameters were omitted by some studies, was another limitation and precluded reaching firm results.

## Conclusions

The preoperative diagnosis of MCT remains controversial. The present study highlights the importance of CA 19-9 as a biomarker in diagnosing MCT, and more specifically, as supported by meta-analysis, it could raise a high degree of clinical suspicion for the early recognition of torsion and early surgical management due to complications related to increased size. Even though elevated CA 19-9 level could not solely differentiate MCT from malignant ovarian masses, no further preoperative investigation is required in cases with ultrasound characteristics of MCT and an elevated biomarker. Nonetheless, the diagnostic value of CA 19-9 is still limited and CA 19-9 can serve only as a supplementary diagnostic tool in patients with MCTs. Further studies are needed in this field to elucidate the exact role of the biomarker and the pathophysiologic pathways of the elevation of CA 19-9.
